# Bioremediation of Artificial Diesel-Contaminated Soil Using Bacterial Consortium Immobilized to Plasma-Pretreated Wood Waste

**DOI:** 10.3390/microorganisms7110497

**Published:** 2019-10-28

**Authors:** Ravit Farber, Alona Rosenberg, Shmuel Rozenfeld, Gabi Benet, Rivka Cahan

**Affiliations:** 1 Department of Chemical Engineering and Biotechnology, Ariel University, Ariel 40700, Israel; ravitf@ariel.ac.il (R.F.); shmuelro@ariel.ac.il (S.R.); 2 Dead Sea-Arava Science Center, Arava 86910, Israel

**Keywords:** bioremediation, bioaugmentation, biofilm, diesel, contaminated soil

## Abstract

Bioaugmentation is a bioremediation option based on increasing the natural in-situ microbial population that possesses the ability to degrade the contaminating pollutant. In this study, a diesel-degrading consortium was obtained from an oil-contaminated soil. The diesel-degrading consortium was grown on wood waste that was plasma-pretreated. This plasma treatment led to an increase of bacterial attachment and diesel degradation rates. On the 7th day the biofilm viability on the plasma-treated wood waste reached 0.53 ± 0.02 OD 540 nm, compared to the non-treated wood waste which was only 0.34 ± 0.02. Biofilm attached to plasma-treated and untreated wood waste which was inoculated into artificially diesel-contaminated soil (0.15% g/g) achieved a degradation rate of 9.3 mg day^−1^ and 7.8 mg day^−1^, respectively. While, in the soil that was inoculated with planktonic bacteria, degradation was only 5.7 mg day^−1^. Exposing the soil sample to high temperature (50 °C) or to different soil acidity did not influence the degradation rate of the biofilm attached to the plasma-treated wood waste. The two most abundant bacterial distributions at the family level were *Xanthomonadaceae* and *Sphingomonadaceae*. To our knowledge, this is the first study that showed the advantages of biofilm attached to plasma-pretreated wood waste for diesel biodegradation in soil.

## 1. Introduction

The motivation for our study was an oil-spill disaster that occurred in December 2014, next to the Evrona Nature Reserve in southern Israel. An oil pipeline a few kilometers away was breached, releasing 5000 m^3^ of crude oil into the reserve. During the remediation activities, an older spill was found 3 km further south, traced back to 1975 and holded 8000–10,000 cubic meters of contamination; for unknown reasons it had been ignored. About a third of the 1975 spill is estimated to still be present, enriching the soil with oil-degrading microorganisms. Oil contamination can be naturally biodegraded with indigenous oil-degrading bacteria. However, enrichment of those in-situ bacteria with exogenous oil-degrading bacteria may be a key factor for increasing bioremediation rates.

Diesel fuel is a fraction of crude oil distilled between 200 °C and 350 °C. It includes a mixture of aromatic compounds, alkanes, cyclic alkanes, and branched alkanes that contain C_9_-C_25_ per molecule. Accidental spills or leakage from pipelines and storage tanks often cause environmental contamination [1]. Diesel fuel is also considered highly toxic and carcinogenic [2]. Technologies for remediation of oil-contaminated soil are based on chemical, physical, and biological approaches. Chemical and physical methods may, however, create another kind of pollution, and they are often costly to implement [3]. Bioremediation is a biological approach considered to be an environmentally friendly and cost-effective alternative to physical and chemical treatments. Bioremediation technology is based on the ability of microorganisms to utilize toxic compounds (such as polychlorinated biphenyls, polycyclic aromatic hydrocarbons, phthalate esters, nitroaromatic pesticides, and petroleum hydrocarbons) as a source of carbon and energy. There are specific microorganisms or microbial consortia that can mineralize pollutants to CO_2_ and H_2_O [4,5,6,7,8].

Bioaugmentation is a bioremediation option based on increasing the natural in-situ microbial population in the contaminated environment [9]. The effectiveness of bioaugmentation is the subject of controversy. Successful petroleum hydrocarbon bioremediation using bioaugmentation was previously reported [10,11,12,13]. For example, Teng et al. (2010) showed that inoculation of *Paracoccus* sp. strain HPD-2 in soil contaminated by polycyclic aromatic hydrocarbons (PAHs) resulted in a 23.2% decrease in total PAH concentrations after 28 days (from 9942 to 7638 μg kg^−1^ in dry soil). The bioaugmented soil exhibited higher enzymatic activity and microbial biomass of the PAH-degrading bacteria. The clear differentiation of the enzymatic activities (*p* < 0.05) between the bioaugmented and control microcosms suggests that the PAH-degrading bacteria were restored in the bioaugmented soil [12].

However, other researchers reported the failure of exogenous microbial in-situ adaptation, described by a decline of the microbial concentration within a short time. The suggested explanation is that the microbial biomass produced in the lab and transferred to the contaminated site suffered from stress when introduced to the natural biotic and abiotic factors in the habitat [14,15,16,17]. Biodegradation of a mixture of PAHs was examined in soil with and without bioaugmentation, using individual bacteria, fungi, and a fungal consortium. In all cases, the low-molecular-weight hydrocarbons (naphthalene, anthracene, and phenanthrene) showed a rapid removal rate, without significant effect on the bioaugmentation. The same phenomenon was observed with the high-molecular-weight hydrocarbons (pyrene, benz[a]pyrene, and benz[a]anthracene). Bioaugmented soil with the isolated *Aspergillus* sp. resulted in a significant increase of benz[a]anthracene and benz[a]pyrene removal. This suggests that *Aspergillus* sp. may have some potential as a bioaugmentation agent [16].

In our study, we tried to overcome the stress on the bacterial consortium upon introduction into the contaminated soil by adding the bacteria as biofilm on wood waste instead of planktonic bacteria. Biofilms are microbial cells attached to surfaces that communicate through quorum-sensing signals and ease their adjustment to the environment [18]. A bacterial biofilm resists mechanical stress [19] and hydration [20], and can survive in nutrient-deficient conditions [21]. The bacterial attachment to a surface is the first step in biofilm formation. The topography and wettability of the surface, the bacterial cell-surface charges, and structures such as pili, fimbriae, flagella, and capsules, all influence the bacterial attachment [22,23,24]. A solid matrix for biofilm formation in bioremediation includes: Ca-alginate [25], activated carbon [26], a polyacrylonitrile membrane [27], geo-textile sheets [6], coal bottom ash [28], and wood waste [24].

In this study, a diesel-degrading consortium was obtained from soil contaminated in the 1975 oil spill found in the Evrona Nature Reserve (located in southern Israel). The obtained diesel-degrading bacteria may be used for further increasing bioremediation rates of the second oil spill in the same reserve. Bioaugmentation of individual bacteria in a bacterial consortium may be limited by the indigenous bacteria and by the in-situ chemical and physical conditions. We assume that bioaugmentation of biofilm on a substratum may overcome these limitations. In this study, wood waste, a cost-effective and natural material, served as the substratum for the diesel-degrading biofilm.

In a previous study published recently, we showed that exposure of wood waste to cold low-pressure nitrogen plasma led to an increase of the surface wettability and probable surface area, resulting in enhanced bacterial attachment and toluene degradation rates [24]. In the present study, the obtained bacteria were grown on plasma-pretreated wood waste in a mineral medium containing diesel as the sole carbon source. The wood waste with the covered biofilm was mixed with Hamra soil artificially contaminated with diesel fuel. The diesel biodegradation in the contaminated soil was evaluated in the presence of the biofilm grown on plasma-pretreated and nontreated wood waste, as well as planktonic bacteria. To our knowledge, this is the first study that shows the advantages of biofilms attached to plasma-pretreated wood waste for diesel biodegradation in soil.

## 2. Materials and Methods 

### 2.1. Preparation of Diesel Emulsion in Mineral Medium 

The preparation of diesel emulsion in mineral medium (MM) [8] or water was performed using a homogenizer ULTRA-TURBAX, T18 basic, (IKA, Königswinter, Germany) equipped with a high speed. The diesel and water were warmed up to 50 °C followed by homogenization for 5 min. The emulsion was prepared immediately before each experiment 

### 2.2. Acquisition of a Diesel-Degrading Microbial Consortium from Oil-Polluted Soil

Soil samples were collected from the site of an old (1975) terrestrial oil spill located at the margins of the Evrona Nature Reserve in southern Israel (29.6685N/34.99210E). Samples were collected aseptically from a depth of ~10 cm.

The collected soil was sifted in a strainer (pores of 1 × 1 mm) and 1 gr soil was suspended in 100 mL of mineral medium (MM) supplied with 0.5% (*v*/*v*) diesel (MMD), followed by incubation at 30 °C with shaking at 170 rpm. After 14 days, the culture reached 0.8 OD 590 nm. It was then diluted to 0.2 OD 590 nm in MMD, using a GENESYS 10S UV-Visible Scientific spectrophotometer (Thermo, Madison, WI, USA), and was grown for another cycle. Portions of the culture with the enriched diesel-degrading bacteria (evidenced by a greenish color) were frozen at −80 °C in 20% glycerol.

### 2.3. Measuring Diesel Concentration with Gas Chromatography−Flame Ionization Detector (GC−FID) Analysis

Headspace sampling was performed with an autosampler, COMBI-xt, CTC Analytics, (PAL SYSTEM, Zwingen, Switzerland). The headspace oven temperature was set at 140 °C, while the loop and transfer line temperatures were set at 150 °C. Vial equilibration time was 20 min. The gas chromatography−flame ionization detector (GC−FID) analysis was performed using a Master GC−FID (DANI, Cologno Monzese, Italy). Separations were performed on a 30 m 0.53 mm column (RTX-5). The injector temperature was 200 °C and the detector temperature was 340 °C. The injection port was operated at a 1:6 split. The flow rate of the carrier gas (helium) was set at 5.0 mL min^−1^. Analyses were performed with an initial column temperature of 45 °C held for 3 min, followed by heating to 275 °C (heating at a rate of 12 °C min^−1^), and holding at 275 °C for 12 min. Total run time was 34.16 min. The diesel fraction contained many peaks (about 18), and all the areas under the whole peaks were taken into account for calculating diesel degradation rates.

### 2.4. Exposure to Cold Low-Pressure Nitrogen Plasma 

The wood waste was exposed to a radiofrequency (RF, 13.56 MHz) inductive-pressure nitrogen plasma cleaner discharge, PDC-3XG, MMT Corp, (Harrick, Ithca, NY, USA). The low-pressure discharge was sustained by an RF oscillating electric field, generated in the gas region at a pressure (P) of ca. 1.0 Torr and a power of 18 W for 1 min. Nitrogen (99.999%) was supplied by Oxygen and Argon Works Ltd., Israel [29].

### 2.5. Biofilm Formation on the Wood Waste 

Diesel-degrading bacteria were grown to log phase in MMD at 30 °C with shaking at 170 rpm. The cultures were diluted to 0.2 OD in a 100 mL bottle containing 10 chips of wood waste from a local pine tree (pieces of 0.06 ± 0.002 g, length of 5 ± 0.5 mm and diameter of 3 ± 0.3 mm), followed by incubation at 30 °C in a static mode for 7 days, except where otherwise noted.

### 2.6. Measurements of the Biofilm Viability 

The viability of the bacteria in the biofilm was analyzed using a colorimetric assay based on a reduction of the reagent 3-(4,5-dimethylthiazol-2-yl)-2,5-diphenyltetrazolium bromide (MTT) by the bacterial hydrogenases. When this reagent is reduced, its color turns from yellow to blue. Thus, viable bacteria in the biofilm turned to a blue color. The next step was dissolving the reduced MTT inside the bacteria by adding dimethyl sulfoxide (DMSO):EtOH (1:1), resulting in a blue solution. The intensity of this solution (proportional to the bacterial hydrogenase activity and bacterial number) was measured using a spectrophotometer at 540 nm.

The wood waste with the attached biofilm (aged 7 days unless otherwise indicated) was gently soaked in phosphate-buffered saline (PBS) in order to remove the planktonic bacteria that was weakly attached to the biofilm. This step was followed by inserting the wood waste with the attached biofilm into a tube containing 2 mL MTT solution (5 mg mL^−1^ of MTT in 0.1 M PBS, pH 7.4) and incubating at room temperature (24 °C) in the dark for 2 h. Then the wood waste was gently washed with PBS (by now the biofilm was colored blue). The reduced MTT in the bacteria was dissolved by adding 2 mL dimethyl sulfoxide (DMSO):EtOH (1:1) for 20 min. The absorbance of the solution was examined using a spectrophotometer at 540 nm [28]. When the absorbance was higher than 1 OD, the sample was diluted and re-examined.

### 2.7. Measuring the Turbidity of the Bacteria in the Biofilm Attached to the Wood Waste

To evaluate the live bacterial amount in the biofilm that was attached to the wood waste, the turbidity (at 590 nm) of the bacteria detached from the biofilm was examined. The bacterial amount in the biofilm was important for the experiments of diesel biodegradation by planktonic bacteria. This served as a planktonic control for comparing the diesel-degrading activity of the bacteria in the biofilm attached to the wood waste.

The plasma-pretreated wood waste with the attached biofilm was inserted into a tube containing MM (10 chips in 15 mL) and placed in a sonication bath for 10 min. The optical density of the detached bacteria in MM was found to be 0.8 ± 0.05 OD 590 nm. It is important to note that the bare wood waste (10 chips, immediately after the bath sonication) was found by MTT analysis to have an optical density of 0.3 ± 0.02 at 540 nm, similar to the original wood waste which was 0.25 ± 0.02. This result indicated that all the live bacteria were detached from the biofilm. In addition, the turbidity at 590 nm of the MM after sonication of the original wood waste (not used for biofilm formation) was 0.02 OD, indicating no release of wood particles to the solution during sonication. Thus, the optical density was only of detached bacteria. Thus, 15 mL of fresh planktonic bacteria (0.8 ± 0.05 OD 590 nm) were added to diesel-contaminated soil and served as a control to diesel degradation by biofilm on 10 chips of wood waste.

### 2.8. Preparation of Artificially Diesel-Contaminated Soil for Biodegradation Experiments 

Hamra soil (a red, sandy clay loam common in Israel) was sifted in a strainer (pores of 1 x 1 mm), and a portion of soil (100 g, pH 6.8) was mixed (using a household mixer) with diesel emulsion for 5 s, to prepare 0.15% and 0.6% diesel in 100 g soil (g/g). The soil was added to 50 mL tubes (the tubes were cut at the bottom to allow irrigation every 3 days). Each tube held 10 chips of wood waste (plasma-treated and non-treated) covered with a 7-day old biofilm. In addition, tubes containing diesel-contaminated soil were inoculated with planktonic bacteria of the same concentration as the biofilm, on 10 chips. The diesel biodegradation in the artificially contaminated soil took place during 2 weeks at 37 °C. To examine diesel evaporation and degradation by the indigenous bacteria, soil was mixed with the diesel emulsion without the addition of the diesel-degrading consortium.

When the Hamra soil was adjusted to pH 5 and 8.0, appropriate aliquots of 1 M HCl and Na_2_CO_3_ (0.01 M) were added into the soil [30]. Measurement of the soil acidity was performed in water using a pH meter. Air-dried Hamra soil (2 g contaminated with 0.15% diesel) was inserted into a beaker and 20 mL of double deionized water were added. The suspension was stirred intermittently for 30 min, followed by standing for about 1 h, at room temperature. Then, the electrode was placed into the clear supernatant, and the pH was recorded once the reading became stable.

### 2.9. Microbial Community Based on 16S rRNA Analysis

The microbial community analysis of the original oil-contaminated soil, as well as the biofilm attached to the plasma-treated and untreated wood waste (prepared as described above), was performed by HyLabs Pvt Ltd., Israel. DNA was extracted with the DNeasy Powersoil kit (Qiagen), operated according to the manufacturer’s instructions. A 16s library preparation for sequencing on Illumina was performed using a 2-step PCR protocol. In the first PCR, the v4 region of the 16S rRNA gene was amplified using the 16s 515F and 806R from the Earth Microbiome Project with CS1 and CS2 tails. The second PCR was done using the Fluidigm access array primers for Illumina, to add the adaptor and index sequences. Sequencing was done on the Illumina Miseq, using a v2-500 cycles kit to generate 2 × 250 paired-end reads. Demultiplexing was performed on Basespace (the Illumina cloud), to generate FASTQ files for each sample. The data was furthered analyzed using CLC Bio to generate operational taxonomic unit (OTU) and Abundance tables. The data for each sample were trimmed for quality and adaptor read-through sequences. The trimmed reads were merged and trimmed to a fixed length. The reads were then subjected to OTU picking using the Greengenes database, to generate the OTU and Abundance tables.

### 2.10. Statistics 

Data are expressed as means  ± SD (standard deviation) of two different experiments and three replicates. The results were statistically analyzed using one-way analysis of variance (ANOVA).

## 3. Results and Discussion

The research hypothesis is that diesel-degrading soil bacteria may become enriched during a prolonged period of diesel contamination and that they can be used for enhancing diesel degradation in a recently contaminated area. In addition, biofilm offers an advantage over planktonic bacteria for bioremediation in extreme environments and high organic matter concentrations. Wood waste is organic matter that can serve as a substratum for diesel-degrading bacteria, as well as increasing soil aeration.

Recently in previous research, we showed that plasma treatment led to enhancement of the wood-waste surface hydrophilicity, which was measured by the apparent contact angle of water. SEM analysis of the plasma-treated wood waste showed a significant change in the internal surface topography. Wood waste that was exposed to plasma led to a 3.5-fold increase in biofilm viability of the exogenously added *P. putida* F1 in MM supplied with toluene, and a 1.6-fold increase in MM supplied with glucose, compared to the untreated wood waste. Similar results were obtained with *B. cereus*. The biofilms of *P. putida* and *B. cereus* grown on wood waste pretreated with plasma led to an increase of toluene degradation, compared to biofilms grown on untreated wood waste [24].

In the current study, diesel (a fraction of crude oil) was chosen for isolating a bacterial consortium from the soil of a 1975 oil spill. The obtained diesel-degrading bacteria may be further used for increasing bioremediation rates of a second oil spill in the Evrona Nature Reserve. The technology of plasma-treated wood waste for biofilm formation can be implemented for bioremediation of that diesel-contaminated soil or other pollutant with suitable biofilm.

### 3.1. Bacterial Consortium Growth Curve in MM with Diesel as the Carbon Source

**Figure 1:**
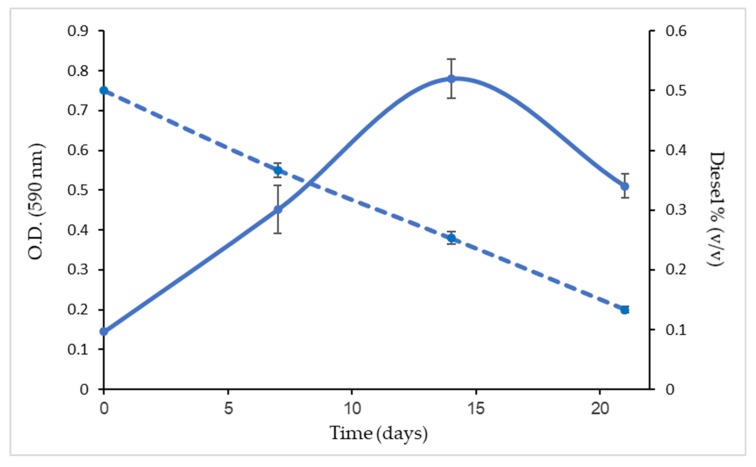
The obtained diesel-degrading consortium growth curve in mineral medium (MM) supplied with 0.5% (*v*/*v*) diesel (MMD).

The obtained bacterial consortium (0.15 OD 590 nm) was grown in sealed bottles in mineral medium, supplemented with diesel (MMD, 0.5% *v*/*v*) that served as the sole carbon and energy source. A bacterial consortium that was grown in mineral medium without a diesel supplement served as the control. The bacterial consortium was incubated for 3 weeks at 30 °C with agitation at 170 rpm. At regular intervals, the bacterial growth and diesel degradation were determined using a spectrophotometer and GC–FID, respectively. As depicted in Figure 1, the bacterial consortium reached 0.78 ± 0.08 OD 590 nm on the 15th day; a reduction of the OD to 0.5 ± 0.02 was observed at the end of the experiment (probably a result of diesel depletion). At the end of the experiment, the residual diesel was 26% and the degradation rate was 1.6 mg day^−1^. In the control sample, where the bacterial consortium was grown without diesel as the carbon and energy source, the optical density was about 0.15 OD 590 nm, which remained stable until the end of the experiment.

The obtained diesel-degrading consortium was also grown on wood waste, and the biofilm on this substratum was applied to biodegradation of the artificially diesel-contaminated soil. The biofilm showed the potential to overcome harsh environmental conditions, such as extreme temperatures and soil acidity.

Biodegradation of environmental pollutants in soil is a slow process, influenced by abiotic factors and by a low abundance of indigenous pollutant-degrading microorganisms. Nutrients and oxygen supplementation, adjustment of pH and temperature (biostimulation), and microbial inoculation (bioaugmentation) were reported to increase biodegradation rates [11,31,32,33]. Bioaugmentation depends on the exogenously added microorganisms, their tolerance to the pollutant, and their capacity to survive in the polluted environment. There are various strategies of bioaugmentation: the use of a single strain [34], a microbial consortium [35], and genetically engineered microorganisms [36]. Nwankwegu et al. (2017) evaluated TPH (total petroleum hydrocarbon) degradation in polluted soil with two different microorganisms, *Micrococcus luteus* and *Rhizopus arrhizus,* as well as with a consortium of both microorganisms. After 8 weeks, the TPH removal was 75.70%, 71.10%, and 66.40%, respectively. It is important to note that there was no statistical difference among the bioaugmentation options [37]. Poi et al. (2017) reported on a commercial-scale bioremediation of petroleum-contaminated soil using bioaugmentation treatment that included 22 bacterial strains. Laboratory experiments showed complete petroleum biodegradation within 21 days. Field experiments with initial petroleum hydrocarbon concentrations of 26,240, 622,657, and 978,399 mg kg^−1^ in 250 tons of soil resulted in petroleum degradation to less than 1000 mg kg^−1^ in 9, 12, and 17 weeks, respectively [38].

### 3.2. Biofilm Formation on Plasma-Treated and Nontreated Wood Waste

Cell immobilization is a well-known method in biological processes for producing metabolites and for wastewater treatment. It provides a high density of microbial population and long-term bacterial survival, leading to a high biodegradation rate [39,40,41]. Recent research showed that exposing wood waste to cold low-pressure nitrogen plasma led to an increase of the wood-waste surface hydrophilicity. In addition, a significant change in the wood-waste morphology was observed, which indicated probable enhancement of the wood-waste surface area. It was also reported that plasma treatment led to an increase of the bacterial attachment and biofilm formation. Biofilms of *Pseudomonas putida* and *Bacillus cereus* that were grown on plasma-pretreated wood waste led to 91% and 89% toluene degradation, respectively; whereas biofilms that were grown on untreated wood waste led to toluene degradation of only 78% and 58%, respectively [24].

**Figure 2:**
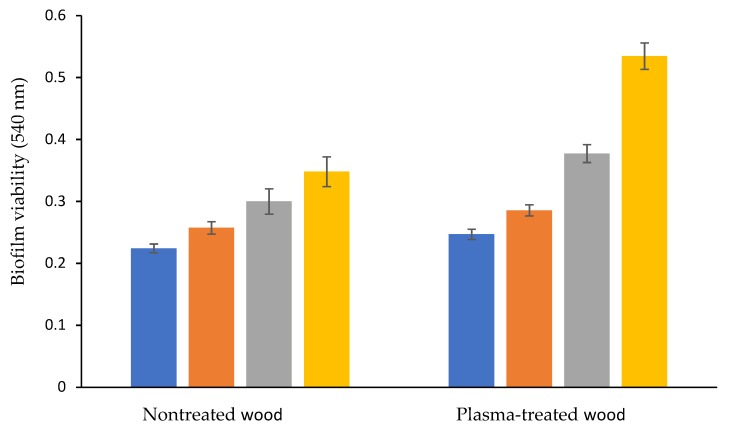
Biofilm viability on plasma-treated (**right**) and nontreated (**left**) wood waste. Measured on the 2nd (blue), 3rd (orange), 4th (grey), and 7th (yellow) day.

In our current research, wood waste was plasma-treated in order to use it as a carrier for biofilm to degrade diesel in contaminated soil. Examination of biofilm formation by the obtained diesel-degrading bacterial consortium on plasma-treated and nontreated wood waste was conducted for 7 days. Ten chips of wood waste were inserted into a bottle containing a suspension of the obtained consortium (0.2 OD 590 nm) in MMD supplied with 0.5% diesel (*v*/*v*) as the sole carbon source. The biofilm viability was examined using MTT analysis. In this assay, the hydrogenases of the diesel-degrading bacteria reduced the MTT salts, and the obtained purple solution was measured by a spectrophotometer (Figure 2). Control samples of treated and nontreated wood waste in sterile MMD served to examine the reaction of the wood waste itself with the MTT salts. The optical density of these controls was reduced from the experimental samples shown in Figure 2.

As can be seen, on the 7th day the biofilm viability on the plasma-treated wood waste reached 0.53 ± 0.02 OD 540 nm, compared to the nontreated wood waste which was only 0.34 ± 0.02. Significantly higher viability on the plasma-treated wood waste, compared to the nontreated, was observed on the 4th and 7th days (*p* < 0.05).

Several methods have been suggested to increase diesel biodegradation rates in water source using immobilized or entrapped bacterial cells. Diesel-degrading microorganisms (*Gordonia alkanivorans* and *Rhodococcus erythropolis*) were immobilized in an entrapped matrix which included alginate and polyurethane–polyurea copolymer, with the addition of active carbon to strength the matrix structure. It was reported that under phosphorous-sufficient conditions, the TPH degradation was 80%. Repeatable batch experiments indicated that the immobilized diesel-degrading bacteria were effective in seawater and groundwater for about 360 days [42]. Zeolites were used for biofilm formation and petroleum-hydrocarbon biodegradation of contaminated water. Biofilms grown on natural zeolite and ammonium-loaded zeolite (where NH_4_ was slowly released and served as the nutrient for the attached biofilm) were compared for diesel biodegradation efficacy. It was reported that the biofilm on the natural zeolite led to a decrease of TPH concentration, from 2.6 to 0.7 mg L^−1^ passing between 20 and 200 bed volumes. In contrast, with the ammonium-loaded zeolite the exit stream was below 0.2 mg L^−1^ (passing 72 to 432 bed volumes), demonstrating higher TPH degradation in the presence of NH_4_ release [43]. The species *Pseudomonas* sp. and *Brevundimonas* sp. were isolated from oil-contaminated seawater and were immobilized on various surfaces (expanded perlite, expanded graphite, and bamboo charcoal). The advantage of biofilm in extreme conditions of salinity was showed by Wang et al., (2015). There, the oil removal rate of the immobilized bacteria was about 85% within 6 days. In addition, the immobilized bacteria displayed good salinity tolerance, compared with the planktonic bacteria [44].

### 3.3. Diesel Concentration in Artificially Contaminated Soil 

**Figure 3:**
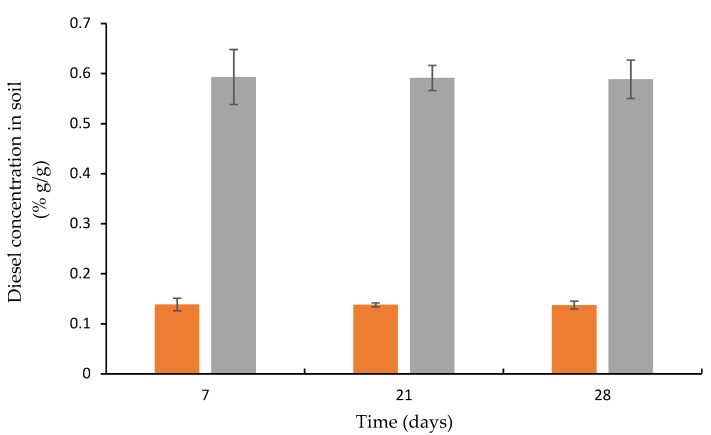
Diesel concentration in artificially contaminated soil: 0.15% (orange) and 0.6% (blue), % (g/g).

Diesel emulsion was prepared using a homogenizer, followed by mixing it with Hamra soil (0.15% and 0.60% diesel in 100 g soil) as described in Materials and Methods. Tubes with the artificially contaminated soil (100 g) were partially sealed and incubated at 37 °C; the residual diesel was examined for 4 weeks. As can be seen in Figure 3, soil samples that were contaminated with 0.15% diesel remained at this concentration until the fourth week. Similar results were obtained for the higher diesel concentration of 0.60%. In addition, when the soil was contaminated with 0.15% and 0.60%, the ratio between the different concentrations was maintained during the four weeks of the experiment. The maximum standard deviation was ±0.1. The Hamra soil was collected from the university area and had not been previously exposed to oil components. Thus, we assume that the indigenous bacteria did not possess diesel biodegradation capabilities, at least within the four weeks of the experiment.

It is important to note that when the soil was contaminated with a suspension of diesel that was not homogenized, the diversity of results was enormous. There was no correlation between the different initial diesel concentrations; moreover, different diesel concentrations were found in different places in the same sample of contaminated soil (data not shown).

### 3.4. Diesel Concentration in Artificially Contaminated Soil Inoculated with Biofilm Attached to Plasma-Treated and Nontreated Wood Waste 

**Figure 4:**
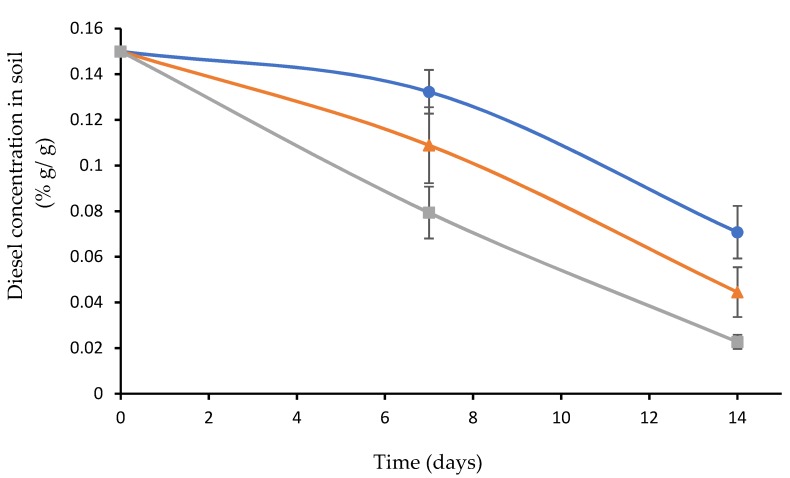
Diesel concentration when the contaminated soil was incubated at 37 °C and inoculated with biofilm attached to plasma-treated wood waste (grey), biofilm attached to untreated wood waste (orange), and planktonic bacteria (blue).

Artificially diesel-contaminated soil was gently mixed with 10 chips of plasma-treated wood waste covered with biofilm. The same procedure was performed with biofilm attached to untreated wood waste. A control of diesel-contaminated soil was inoculated with a fresh planktonic bacterial consortium (15 mL) with the same optical density (0.8 OD 590 nm) of the detached bacteria of the plasma-treated wood waste. Another two controls of plasma-treated and untreated wood waste were prepared for examination of the diesel absorbed by the wood waste itself (10 chips in 100 g diesel-contamination). All the experimental and control samples (each with 4 replicates) were incubated at 37 °C for 4 weeks. The residual diesel was analyzed with a GC–FID device (Figure 4).

The results showed that when the soil was inoculated with biofilm attached to plasma-treated wood waste, the residual diesel was only 13.3% with a degradation rate of 9.3 mg day^−1^. When the soil was inoculated with biofilm attached to the untreated wood waste, the residual diesel was 26.7% with a degradation rate of 7.8 mg day^−1^. In the soil samples inoculated with the planktonic bacteria, the residual concentration was much higher (46.7%) with a degradation rate of only 5.7 mg day^−1^. These results indicate that the biofilm of the diesel-degrading consortium on the plasma-treated wood waste led to the highest diesel degradation rate. It is important to note that 5% of the diesel in the soil was absorbed into the wood waste itself (plasma-treated as well as untreated). The diesel concentration in the soil that was inoculated with the biofilm includes the 5% of the diesel absorbed by the wood waste. We assume that this parameter facilitated the diesel degradation and contributed to the degradation rate.

### 3.5. Diesel Biodegradation as a Function of a Pre-Exposure to High Temperature

**Figure 5:**
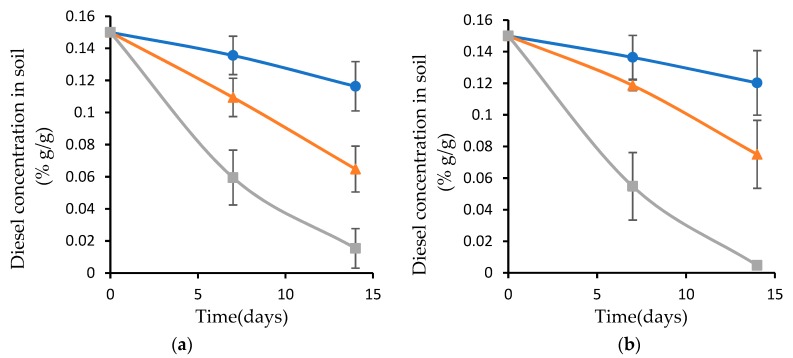
Diesel concentration in the inoculated soil pretreated at 50 °C for 4 h (**a**), and 6 h (**b**), followed by incubation at 37 °C for the rest of the experiment. Soil inoculated with biofilm attached to plasma-treated wood waste (grey); biofilm attached to nontreated wood waste (orange); and planktonic bacteria (blue).

The soil samples with the inoculated biofilm and planktonic bacteria were pretreated by exposure to 50 °C for 4 and 6 h, followed by incubation at 37 °C for the rest of the experiment (Figure 5a,b).

**Figure 6:**
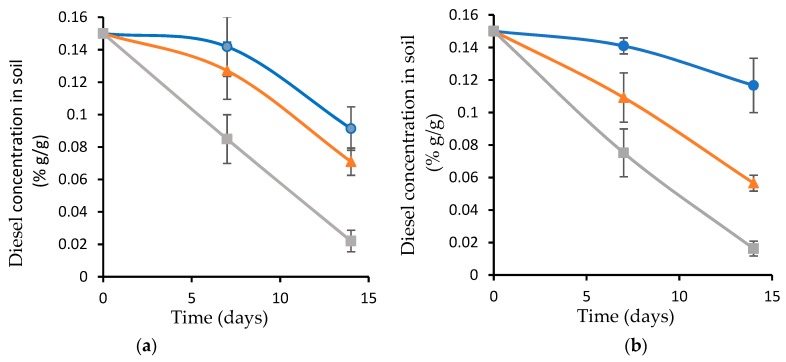
Diesel concentration as a function of soil acidity, pH 5 (**a**) and pH 8 (**b**). Soil that was inoculated with the plasma-treated wood waste covered with biofilm (grey); untreated wood waste covered with biofilm (orange); and planktonic bacteria (blue).

When the inoculated soil was exposed to 50 °C for 4 h (Figure 5a) and 6 h (Figure 5b), the diesel degradation rate by the biofilm attached to the plasma-treated wood waste was about the same as when the experiment was conducted at 37 °C. The biofilm on the untreated wood waste led to a degradation rate of 6.4 mg day^−1^ and 5 mg day^−1^ when the experiment was performed at 50 °C for 4 and 6 h, respectively. The degradation rate in the presence of planktonic bacteria was only 2 mg day^−1^ in both exposure duration times.

From these results it can be seen that the biofilm on plasma-pretreated wood waste led to the highest diesel biodegradation at all experimental temperatures, and that the degradation rate was not affected by the high temperature. The degradation rate of the biofilm on the untreated wood waste was minimally influenced by high temperature. This phenomenon can be explained by the fact that the bacteria in the biofilm are surrounded by a matrix containing extracellular polymeric substances (EPS), which provide resistance to high temperatures. In contrast, the planktonic bacteria were more sensitive to high temperature. The higher resistance of the biofilm that was attached to the plasma-pretreated wood waste, compared to the biofilm that was attached to the untreated wood waste, can be explained by the higher bacterial amount (Figure 2) and the relative distribution (Table 1). However, the planktonic bacterial consortium was highly affected by exposure to high temperatures, and its diesel-degrading rate was about 4.7-fold less, compared to the biofilm on the plasma-treated wood waste.

### 3.6. Diesel Biodegradation as a Function of Soil Acidity 

The diesel-contaminated soil was adjusted to pH 5 and pH 8, and inoculated with the plasma-treated and nontreated wood waste covered with the biofilm, followed by incubation at 37 °C. A soil sample inoculated with the planktonic bacteria served as a control. The diesel biodegradation rates in the different pH values (Figure 6a,b) were compared to the degradation rate in the original, with pH 6.8 (Figure 4).

The biofilm attached to the plasma-treated wood waste that was inoculated in the soil adjusted to pH 5, or to pH 8, led to a diesel degradation rate of 9.3 mg day^−1^, the same as with the original soil (pH 6.8). The biofilm on the untreated wood waste that was inoculated in soil adjusted to pH 5 and pH 8 led to a slight change in the degradation rate compared to the original soil: 5.7 mg day^−1^ and 6.5 mg day^−1^, respectively. The planktonic bacteria yielded a diesel degradation rate of only 4.3 mg day^−1^ at pH 5, and 2.1 mg day^−1^ at pH 8, while in the original soil (pH 6.8) it was 5.7 mg day^−1^.

To summarize, the diesel-degrading consortium was obtained from the desert, where soil temperatures in the summer can reach at least 44 °C for several hours a day. When the planktonic bacteria were exposed to 50 °C for 4 and 6 h, a reduction in the diesel degradation rate was observed. However, the biofilm on the wood waste preserved its diesel-degrading activity even when the soil was exposed to 50 °C for 6 h (Figure 5). We assume that the difference in the diesel degradation rates between the biofilm grown on the plasma-treated wood waste and the biofilm grown on the untreated wood waste is due to the higher bacterial viability on the plasma-treated wood waste (Figure 2). The biofilm was also less affected by the change in the soil acidity, compared to the planktonic consortium (Figure 6).

Biodegradation of petroleum components was reported in cold as well as hot environments; this was ascribed to the adaptation of the indigenous microorganisms to the environmental temperature. The prevalence of several biodegrading *n*-alkanes, such as *Pseudomonas putida*, *Acinetobacter* spp., *Rhodococcus* spp., and *Mycobacterium* sp., were found in oil-contaminated Alpine soils. A significantly higher percentage of these bacteria was found in the contaminated (50 to 75%) compared to the pristine (0 to 12.5%) soils, confirming enrichment of the biodegrading microorganisms following contamination. A significant positive correlation was reported between the level of TPH contamination and the number of biodegrading *n*-alkanes bacteria. However, only partial correlation was found with the TPH contents [45]. Microorganisms able to degrade polycyclic aromatic hydrocarbons (PAHs) were isolated from the Unkeshwar hot spring in India. The obtained consortium (*Aeribacillus pallidus*, *Bacillusaxarquiensis*, *Bacillus siamensis*, and *Bacillus subtilis* subsp. *inaquosorum*) was found to be thermophilic and thermo-tolerant. This consortium produced a relatively high PAH degradation rate at 50 °C [46].

### 3.7. Microbial Distribution Analysis (Based on 16S rRNA) in the Original Oil-Contaminated Site and in the Biofilms 

The microbial diversity in the original contaminated soil was evaluated based on 16S rRNA. Operational taxonomic unit (OTU) reads were identified and phylogenetically classified. The predominant phylum was *Proteobacteria* (52%), the second-most predominant was *Actinobacteria* (18%), and the third was *Firmicutes* (8%). The other phyla (40) each gave evidence of relatively low abundance. It is important to note the high prevalence of unassigned phyla (about 7%), which may be attributed to either a significant amount of novel species or poorly identified taxonomy. Taxonomic classification at the order level revealed that the 10 most predominant orders were: *Actinomycetales* (16.6%), *Xanthomonadales* (15.4%), *Bacillales* (6.9%), *Sphingomonadales* (5.2%), *Pseudomonadales* (4.6%), *Rhizobiales* (4.2%), *Rhodospirillales* (3.3%), *Oceanospirillales* (3.3%), *Chromatiales* (2.6%), and *Burkholderiales* (2.5%). A total of 189 distinct orders and nearly 300 families were identified. Orders and families with low relative distribution (less than 3%) were not taken into account for the percentage calculations.

**Figure 7:**
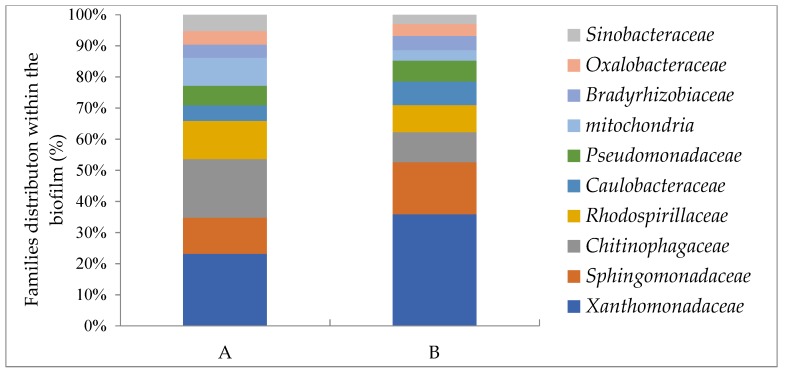
Bacterial distribution of the top 10 at the family level (81% and 67.5%, respectively of all identified families) which were identified in the biofilm grown on the plasma-treated (**A**) and untreated wood waste (**B**).

Plasma-treated and untreated wood waste in MMD were inoculated with the obtained diesel-degrading consortium for 7 days. The bacterial distribution in the biofilm which was grown on the wood waste was analyzed by microbial 16S rRNA. The bacterial distribution at the phylum level in the biofilm grown on the plasma-treated wood waste included a major phylum, *Proteobacteria* (82.8%), and a minor phylum, *Bacteroidetes* (8.3%); the rest (12 phyla) each showed relatively low abundance. The phyla diversity of the biofilm that was grown on the untreated wood waste was *Proteobacteria* (71.5%) and *Bacteroidetes* (13%); the rest (12 phyla) each showed relatively low abundance. The bacterial distribution at the class level in the biofilms grown on the plasma-treated and untreated wood waste included two main classes: **γ**-*Proteobacteria* (40.5% and 30.4%, respectively), and α-*Proteobacteria* (34.4% and 31.7%, respectively). These phyla mainly included oil-degrading bacterial families (9 of the top 10 families): *Xanthomonadaceae*, *Sphingomonadaceae*, *Chitinophagaceae*, *Rhodospirillaceae*, *Caulobacteraceae*, *Pseudomonadaceae*, *Bradyrhizobiaceae*, *Oxalobacteraceae* and *Sinobacteraceae*, except for the *mitochondria* (Figure 7). The families’ relative distribution, and references reporting their oil-degrading abilities, is shown in Table 1.

The bacterial distribution at the genus level in the biofilm attached to the plasma-treated and untreated wood waste was poorly identified. The major genus in the most abundant bacteria for the family *Xanthomonadaceae* was not identified (80%). In this family the genera *Lysobacter*, *Pseudoxanthomonas,* and *Thermomonas* were identified and exhibited very low relative abundance of 20%. In the family *Sphingomonadaceae*, a genus which exhibits relative abundant of 40% was not identified, while two other genera, *Kaistobacter* and *Sphingomonas*, exhibited relative abundance of 60%.

To summarize, the most abundant phylum in the original diesel-contaminated soil was *Proteobacteria* (52%). The abundance of this phylum was accelerated in the biofilm attached to plasma-treated and untreated wood waste that was grown in MM supplied with diesel: 82.8% and 71.5%, respectively. The bacterial distribution at the family level showed that in the biofilm, the two most abundant were *Xanthomonadaceae* and *Sphingomonadaceae*. In the biofilm attached to the plasma-treated wood waste *Xanthomonadaceae* was 29%, while on the untreated wood waste it was 16%; in the original soil *Xanthomonadaceae* was only 4%. The second most abundant family in the biofilm attached to plasma-treated and untreated wood waste was *Sphingomonadaceae*, 13% and 8%, respectively.

An interesting finding showed a significantly higher distribution of diesel-degrading bacteria in the biofilm attached to the wood waste, compared to that found in the original soil (cited studies in Table 1). Nine of the 10 families which were found in the biofilm were reported to exhibit hydrocarbon biodegradation activity. We assume that the bacterial ability of biofilm formation also influenced on their relative distribution in the biofilm.

**Table 1:** Bacterial relative distribution at the family level of the top 10, relative to all identified families in the original diesel contaminated soil, within the biofilm grown on the plasma-treated and untreated wood waste.

Families	Soil (%)	Biofilm on the Plasma-Treated Wood Waste (%)	Biofilm on the Untreated Wood Waste (%)	Hydrocarbon Activity Reported Previously
*Xanthomonadaceae*	4	29	16	[47,48]
*Sphingomonadaceae*	3	13	8	[49,50]
*Chitinophagaceae*	0.29	8	13	[51]
*Rhodospirillaceae*	1	7	8	[52,53]
*Caulobacteraceae*	2	6	3	[52]
*Pseudomonadaceae*	4	6	4	[48]
*Bradyrhizobiaceae*	0.04	4	3	[53]
*mitochondria*	0.22	3	6	
*Oxalobacteraceae*	0.04	3	3	[47]
*Sinobacteraceae*	11	2	2	[54]
sum	26.09%	81%	66%	

Prince et al. (2010) reported on hydrocarbon-degrading bacteria classified at the phylum level as *Proteobacteria*, *Actinobacteria*, *Firmicutes*, *Bacteroidetes*, *Chlamydiae*, and *Deinococcus*-*Thermus* [55]. In other studies, it was reported that hydrocarbonoclastic bacteria belonging to the phyla *Actinobacteria*, *Proteobacteria*, and *Firmicutes* have been widely distributed in oil-contaminated soil [56,57]. A significant increase in the relative distribution of *Proteobacteria* occurred in diesel-polluted soils after the second week, whereas other phyla decreased, especially *Actinobacteria* [57]. Bell et al. (2013) reported that *Actinobacteria* and *Proteobacteria* were dominant in hydrocarbon-contaminated Arctic soils [47].

Popp et al. (2006) previously reported on the predominance at the class level of *γ- Proteobacteria* in hydrocarbon-contaminated soil [58]. The same observation was found in petroleum refinery sludge [51]. Jung et al. (2014) evaluated red clay soil as a biostimulation agent in diesel-contaminated soils for diesel biodegradation and bacterial distribution evaluation. It was reported that more than 70% of the total community at the phyla level were *Proteobacteria*, *Acidobacteria*, and *Actinobacteria*. Meanwhile, *Gemmatimonadetes*, *Chloroflexi*, *Nitrospira*, *Bacteroidetes*, *Planctomycetes*, *Firmicutes*, and *Cynobacteria* accounted for approximately 10%. A large portion, about 20%, was accounted as unassigned bacteria. Taxonomic classification at the family level of diesel-contaminated soil indicated increases in *Nocardioidaceae*, *Pseudomonadaceae*, *Xanthomonadaceae*, and *Caulobacteraceae* [48].

In our study, the predominant phylum in the original oil-contaminated soil was *Proteobacteria* (52%). The predominance of this phylum was accelerated to more than 70% in the biofilm which was grown in the presence of diesel, on the plasma-treated as well as untreated wood waste. We assume that the combination of several bacterial strains helped accelerate the biodegradation process.

However, the cited studies showed that other phyla were predominant as a consequence of oil-component contamination. It was further reported that the extent and dynamics of microbial distribution were found to be dependent on the soil type, the hydrocarbon composition, and the pollution time [59]. A drastic environmental disturbance such as a fire or pollution may cause huge changes in the bacterial diversity and its community structure [60].

To summarize, the innovative contribution of this study is that plasma-treated wood waste can serve for biofilm formation and enhance the bioremediation of diesel-contaminated soil. Moreover, this technology can be implemented for other toxic organic pollutants in soil. The advantages of this technology include the substratum for biofilm attachment being a natural material, and the plasma treatment being a clean technology that is not based on chemicals or extreme temperatures.

## 4. Conclusions

A diesel-degradable consortium that was obtained from oil-contaminated soil was grown on wood waste pretreated with plasma. The cold low-pressure nitrogen plasma treatment led to an increase of bacterial attachment and diesel degradation rates. On the 7th day of growth, the biofilm viability on the plasma-treated wood waste reached 0.53 ± 0.02 OD 540 nm, compared to biofilm on the nontreated wood waste (0.34 ± 0.02). Biofilm attached to plasma-treated and untreated wood waste which was inoculated into artificially diesel-contaminated soil (0.15% g/g) achieved degradation rates of 9.3 mg day^−1^ and 7.8 mg day^−1^, respectively. Meanwhile, in the soil inoculated with planktonic bacteria, the rate was only 5.7 mg day^−1^. Exposing the soil sample to high temperature (50 °C) for 4 and 6 h did not influence the degradation rate of the biofilm grown on the plasma-treated wood waste, whereas the degradation rate of the planktonic consortium was reduced three-fold. To our knowledge, this is the first study to show the advantage of biofilms attached to plasma-pretreated wood waste for diesel biodegradation in soil.
