# Erratum: Farber, R.; Rosenberg, A.; Rozenfeld, S.; Banet, G.; Cahan, R. Bioremediation of Artificial Diesel-Contaminated Soil Using Bacterial Consortium Immobilized to Plasma-Pretreated Wood Waste. *Microorganisms* 2019, *7*, 497

**DOI:** 10.3390/microorganisms7120677

**Published:** 2019-12-10

**Authors:** Ravit Farber, Alona Rosenberg, Shmuel Rozenfeld, Gabi Banet, Rivka Cahan

**Affiliations:** 1 Department of Chemical Engineering and Biotechnology, Ariel University, Ariel 40700, Israel; ravitf@ariel.ac.il (R.F.); alonar@ariel.ac.il (A.R.); shmuelro@ariel.ac.il (S.R.); 2 Dead Sea-Arava Science Center, Arava 86910, Israel

The authors wish to make the following erratum in this paper [1].

There is a mistake in the spelling of the fourth author’s name:

Ravit Farber ^1^, Alona Rosenberg ^1^, Shmuel Rozenfeld ^1^, Gabi Benet ^2^ and Rivka Cahan ^1,^*

the corrected spelling should be:

Ravit Farber ^1^, Alona Rosenberg ^1^, Shmuel Rozenfeld ^1^, Gabi Banet ^2^ and Rivka Cahan ^1,^*

The authors apologize for any inconvenience this may have caused.
